# Side Effects of Brolucizumab

**DOI:** 10.18502/jovr.v16i4.9757

**Published:** 2021-10-25

**Authors:** Tahmineh Motevasseli, Saeed Mohammadi, Fatemeh Abdi, William R. Freeman

**Affiliations:** ^1^Ophthalmic Research Center, Research Institute for Ophthalmology and Vision Science, Shahid Beheshti University of Medical Sciences, Tehran, Iran; ^2^Department of Ophthalmology, Shiley Eye Institute, University of California San Diego, La Jolla, CA, USA; ^3^Jacobs Retina Center, University of California San Diego, La Jolla, CA, USA; ^4^Farabi Eye Hospital, Tehran University of Medical Sciences, Tehran, Iran; ^5^Department of Ophthalmology, Abadan University of Medical Sciences, Abadan, Iran; ^6^Eye Research Center, The Five Senses Institute, Rassoul Akram Hospital, Iran University of Medical Sciences, Tehran, Iran

**Keywords:** Anti-vascular Endothelial Growth Factor, Brolucizumab, Wet Age-related Macular Degeneration

## Abstract

Age-related macular degeneration and its complication, subretinal neovascularization, are common causes of progressive, irreversible impairment of central vision. Anti-vascular endothelial growth factor (anti-VEGF) therapy has improved the visual outcome and provided an evolution in the treatment of retinal disease. The current four anti-VEGF drugs – pegaptanib, ranibizumab, aflibercept, and bevacizumab – have been administered for many years. A new anti-VEGF agent, brolucizumab, was approved by the U.S. Food and Drug Administration (FDA) in late 2019 for the treatment of wet age-related macular degeneration. Brolucizumab is a novel single-chain fragment variable antibody that inhibits all isoforms of VEGF-A and has been suggested to have more tissue penetration. Despite all the benefits, there are some reports of serious side effects that need to be understood in managing patients. Brolucizumab has been reported to cause occlusive retinal vasculitis in the setting of intraocular inflammation, which has not been seen in other anti-VEGF medications. A PubMed and Scopus search was performed and all article types were included. In the present article, we have reviewed the reported side effects of brolucizumab.

##  INTRODUCTION

Age-related macular degeneration (AMD) is the leading cause of blindness in the developed countries and is forecasted to affect 288 million individuals by 2040.^[1]^ Pharmacologic treatment of neovascular AMD (nAMD) was first introduced using pegaptanib as an anti-VEGF drug but was then revolutionized by introduction of newer anti-vascular endothelial growth factors (anti-VEGF) such as bevacizumab (Avastin; Genentech, South San Francisco, California), ranibizumab (Lucentis; Genentech, South San Francisco, California), and aflibercept (Eylea; Regeneron, Tarrytown, New York).^[2,3,4]^ However, despite significant advancements, long-term results are less promising, since about 30% of involved eyes will experience significant vision loss due to geographic atrophy in the course of the treatment.^[5,6]^ Considering the burden of repeated injections and consequent lower patient's compliance, the need for newer and more efficient treatments is clear.^[7]^

Brolucizumab (Beovu, Novartis Pharma AG, Basel, Switzerland) is a novel single-chain fragment variable (scFv) antibody that inhibits all isoforms of VEGF-A and prevents binding of this ligand to VEGFR-1 and VEGFR-2.^[8]^ In comparison to a full antibody (bevacizumab) or a Fab fragment (ranibizumab), this 26 kDa humanized antibody is half the size of ranibizumab and may have^[9]^ a relatively easier manufacturing process,^[10]^ and has been suggested to have more tissue penetration with 2.2 and 1.7 times higher exposure in neurosensory retina and retinal pigment epithelium (RPE), respectively^[11]^ due to its smaller size. It likely also has a more rapid elimination half-life which may partially offset the improved penetration. In theory, the small size also results in more rapid renal clearance and theoretically fewer systemic side effects^[12,13]^ due to smaller size. According to HAWK and HARRIER studies, this novel drug has superior anatomic results with greater fluid resolution in wet macular degeneration. It was demonstrated that brolucizumab could achieve a similar best-corrected visual acuity (BCVA) compared to aflibercept with the advantage of the possibility of extension of the regimen to 12-week intervals.^[14]^ Despite all the hopes of starting a new area in the treatment of nAMD, reports of serious side effects have endangered this idea. In this article, we have reviewed the reported side effects of brolucizumab.

##  METHODS

A PubMed and Scopus search was performed in February 2021 using each of the following keywords: “Brolucizumab”, “side effects”, “Eye”, “Ocular”, “HAWK”, and “HARRIER” in different combinations. All article types including original articles, reviews, and case reports that described the side effects of brolucizumab were identified. No limitation for the time of publication was applied. Abstracts only and non-English articles were excluded. All selected articles were reviewed thoroughly by the authors and relevant articles describing the side effects of brolucizumab were discussed.

##  RESULTS

### Intraocular inflammation/Retinal vasculitis

Since February 2020 which was when the initial cases of serious ocular side effects were reported, more have come into light. Eleven and twenty-one cases of occlusive vasculitis were reported in February 23 ${}^{\mathrm{rd}}$ and March 30 ${}^{\mathrm{th}}$ of 2020, respectively, by The American Society of Retina Specialists (ASRS).^[15]^ On March 31, 2020, Huag and colleagues^[16]^ reported an 88 year-old patient who developed painless decrease of vision in association with photosensitivity in both eyes four weeks after bilateral intravitreal brolucizumab due to nAMD. Examination revealed anterior chamber reaction in both eyes with retinal arterial occlusion, vasculitis, and optic nerve inflammation in fluorescein angiography (FA). Intravitreal dexamethasone was implanted in left eye which resulted in improvement of the inflammation. They hypothesized that type IV hypersensitivity reaction is responsible for intraocular inflammation (IOI). Jain and coworkers^[17]^ reported a 92-year-old patient who developed decreased vision in left eye after third intravitreal brolucizumab injection for nAMD. Funduscopy revealed mild vitritis in association with flame-shaped hemorrhage at the superior optic disc margin, retinal whitening surrounding the proximal portion of the supero-temporal branch of the central retinal artery, and intra-arteriolar greyish material in the left eye. FA showed delayed filling of several arterioles in early and late phases without perivasculature or optic disc leakage in the left eye. The patient was closely observed as there was no active inflammation. Baumal and colleagues^[18]^ evaluated 15 eyes of 12 patients with retinal vasculitis and IOI due to intravitreal injection of brolucizumab from 10 centers in United States to identify features and outcomes of this condition. They reported that retinal vasculitis and IOI is mostly diagnosed 30 days after the most recent injection of brolucizumab. They showed that the affected eyes develop focal or elongated segmental sheathing, vascular nonperfusion, sclerotic vessels, cotton-wool spots, irregularity and dilation of veins, perivenular hemorrhages, and foci of phlebitis. FA showed delayed arterial filling, vascular nonperfusion, and dye leakage from the optic nerve and vessels. Steroids were effective in improvement of inflammation and FA findings; however, an improvement in visual acuity was related to the location and severity of retinal occlusive vasculitis. Although, two patients underwent pars plana vitrectomy, no improvement was seen. Witkin *et al.* ^[19]^ evaluated 26 eyes of 25 patients with retinal vasculitis among postapproval cases (the number of postapproval cases is unknown) following the initial study containing 37,000 patients who received over 70,000 injections of Beovu for treatment of nAMD and were reported to the American Society of Retina Specialists (ASRS) until April 1, 2020. Postapproval cases of retinal vasculitis voluntarily reported to the ASRS as of April 1, 2020. First symptoms of intraocular inflammation primarily happened 25 days following the intravitreal injection of brolucizumab. Patients developed decreased vision, floaters, pain, and redness; however, two of them were asymptomatic and were diagnosed of the follow-up examinations. About one third of the patients only had anterior IOI, a third only had posterior IOI, and another third had both anterior and posterior segments inflammation; however, two eyes had no IOI other than retinal vasculitis. Retinal arteries, veins, and choroidal vessels were involved. Optic nerve leakage and choroidal ischemia was also noted on FA. Although, there was no established treatment method, most of the patients received topical corticosteroids, nearly half of the patients received systemic corticosteroids, about fourth of them received intravitreal corticosteroids, and 15% of the patients underwent pars plana vitrectomy. IOI and retinal vasculitis resulted in permanent decrease in vision following resolution of the inflammation.

The FDA has reported a 4% chance of IOI and a 1% chance of retinal artery occlusion.^[20]^ Post-hoc analysis reports from two-year, double-masked, multicenter, active-controlled randomized phase-III clinical trials (HAWK and HARRIER) among 1817 brolucizumab-treated eyes revealed 50 patients with definite/probable drug-related side effects within the spectrum of IOI, retinal vasculitis, and/or vascular occlusion. The incidence of IOI was 4.6%, retinal vasculitis associated with IOI was 3.6%, and retinal vascular occlusion occurred in 2.1% of the patients.^[21,22]^ Sharma *et al.* evaluated 42 patients who were previously treated with anti-VEGF drugs in BREW study. They followed their patients for a mean period of 7.2 ± 3.6 weeks, assessed the occurrence of ocular or systemic adverse events, and reported no case of inflammation, vasculitis, or any other ocular or systemic adverse effects.^[23]^

Narayanan *et al.* ^[24]^ reported a 62-year-old female who previously received 30 intravitreal injections of aflibercept, ziv-aflibercept, ozurdex, and ranibizumab. The patient also underwent reduced fluence PDT in the course of the treatment. The vision deteriorated due to significant subretinal scar, atrophy of outer retinal layers, and cataract. Cataract surgery was performed, and the patient received intravitreal injection of brolucizumab due to persistent intraretinal fluid one month after the previous intervention. One day after the injection, the patient had minimal conjunctival injection, 1 mm hypopyon, without lid edema, and no view of the fundus due to intense vitritis. The patient underwent pars plana vitrectomy and intravitreal injection of ceftazidime and vancomycin. Vitreous sample showed no growth on culture neither any organism on the smear. Polymerase chain reaction for Eubacteria was also negative. Prednisolon acetate 1% was started frequently in association with topical moxifloxacin and homatropine eye drops which resulted in significant improvement of the vision on the fifth day. They hypothesized that along with delayed hypersensitivity reaction which causes retinal vasculitis, immediate hypersensitivity reaction similar to Toxic Anterior Segment Syndrome (TASS) could also happen due to intravitreal injection of brolucizumab. Iyer *et al.* ^[25]^ reported a 76-year-old woman with nAMD who had persistent subretinal fluid and a large pigment epithelial detachment in the right eye despite receiving multiple intravitreal anti-VEGF treatment. The patient received three monthly injection of brolucizumab in the right eye and developed decreased vision, anterior chamber reaction, vitritis, and peripheral vascular sheathing one week after the third injection. This condition was improved by using topical prednisolone acetate 1%. The patient was switched to ranibizumab, one month after the previous brolucizumab injection. She developed decreased vision due to significant occlusive retinitis which was unresponsive to corticosteroid treatment. She underwent pars plana vitrectomy in order to remove the inflammatory mediators in association with intravitreal triamcinolone injection which resulted in mild improvement of vision and signs of retinitis. They suggested that delayed onset inflammatory mechanisms (especially type III) are responsible for IOI due to intravitreal injection of brolucizumab. Kondapalli^[26]^ reported a 77-year-old women who developed retinal vasculitis after second intravitreal injection of brolucizumab. The patient had been previously treated with anti-VEGF drugs. Numerous keratic precipitates, vitreous cells, and retinal vasculitis was revealed in ophthalmic examination and FA showed retinal ischemia with capillary nonperfusion and disc leakage which were consistent with the diagnosis of occlusive retinal vasculitis. The patient was unresponsive to corticosteroids.

Phase-II clinical trial for evaluation of the efficacy and safety of intravitreal brolucizumab showed that the most common adverse effects of brolucizumab is subconjunctival hemorrhage, vitreous floaters, reduced visual acuity, and vitreous detachment. Although upper respiratory tract infection and urinary tract infection were the most commonly reported non-ocular adverse effects, the systemic adverse events were similar in the brolucizumab and aflibercept groups without a significant difference. The incidence of eye inflammation and keratitis was reported to be 2.3% among 44 participants in the study. The other reported adverse events were cataract and macular fibrosis which were seen in both groups and were probably related to the age and progression of AMD.^[27]^ The 48-week report of the HAWK and HARRIER (phase-III clinical trial for the evaluation of the efficacy and safety of intravitreal brolucizumab) studies reported that the most common adverse effects are conjunctival hemorrhage and reduced visual acuity. Incidence of uveitis and iritis were reported to be 2.2% with brolucizumab; 90% of these cases were mild to moderate and treated with a short course of topical corticosteroids without any sequels. Retinal artery occlusion was reported to happen in 0.6% and 0.3% of the participants after 48 weeks in HAWK and HARRIER studies, respectively. Other reported side effects were lenticular opacities, punctate keratitis, corneal abrasion, posterior capsule opacification, cataract, increased intraocular pressure, blepharitis, conjunctivitis, and iritis.^[14]^ The 96-week report of HAWK and HARRIER study showed that the most common adverse effects are conjunctival hemorrhage and reduced visual acuity. Iritis and uveitis were the most frequent inflammatory adverse effects. In HAWK study, iritis occurred in 0.8% and 2.5% of the patients and uveitis occurred in 1.7% and 2.2% of the patients in 3 mg and 6 mg study group, respectively; however, HARRIER study revealed < 1% incidence of iritis and uveitis for 6 mg group. These studies showed that about 50% of IOI events happen in the first 12 weeks after injection. Arterial thromboembolic events occurred in 1.1% and 1.4% of the patients in 3 mg and 6 mg study group of HAWK study, respectively; and in 1.6% of the brolucizumab receiving patients in HARRIER study. Among them, four patients in the 3 mg group and six patients in the 6 mg group had retinal artery occlusion and all of them had cardiovascular comorbidities such as hypertension or cardiac arrhythmias. The other mentioned adverse effects in the study were retinal hemorrhage, cataract, dry eye, eye pain, posterior capsule opacification, increased intraocular pressure, blepharitis, retinal pigment epithelial tear, punctate keratitis, corneal abrasion, conjunctivitis, and macular fibrosis.

##  DISCUSSION

Brolucizumab, a recently approved single-chain fragment variable (scFv) is targeted to VEGF for treatment of nAMD. Its approval was thought to produce better patient care as it has better fluid control with less frequent injection schedule in eyes with nAMD. It was also beneficial for eyes that were nonresponsive to available anti-VEGF drugs. However, the relatively high incidence of IOI particularly retinal vascular inflammation and occlusion has raised safety concerns, since about 30% of the eyes will develop visual loss due to retinal arterial events. Unlike the HAWK and HARRIER studies which reported the incidence of retinal vasculitis with retinal vascular occlusion 2.1%, it was recently reported to be 4.6 per 10,000 injections in the post-marketing surveillance.[28] This difference might be due to difference in recruited population who were treatment naïve in the phase-III clinical trial, since patients who were previously treated with anti-VEGF drugs were more susceptible to IOI. Few points should be noted regarding the clinical presentation of IOI; first is the predilection of IOI to affect arteries more than veins. The other issue is gender predominance; IOI mainly happens in women who had been previously treated with anti-VEGF drugs. IOI has also been reported with other anti-VEGF drugs such as bevacizumab,^[29]^ ranibizumab,^[30]^ and aflibercept.^[31]^ Single-chain fragment variable is the smallest functional part of an antibody and results in a higher molar concentration and higher antigenic load of the drug which may be responsible for the longer duration of action. Anti-drug antibodies (ADA) existed in about 35–50% of the treatment naive eyes in the HAWK and HARRIER trials which could be the reason for antigen-antibody immune complex deposition and subsequent type-III hypersensitivity reaction which is postulated to be reason for IOI and retinal vasculitis.^[32]^

##  SUMMARY

The clinical usefulness of this novel FDA-approved drug for the treatment of nAMD is now being questioned because of the incidence of adverse effects; however, it should be carefully evaluated in order not to lose this treatment option.
