# Erratum: Side Effects of Brolucizumab

**DOI:** 10.18502/jovr.v17i1.10185

**Published:** 2022-01-21

In the article titled “Side Effects of Brolucizumab,” published on pages 670–675, Issue 4, Volume 16 of *Journal of Ophthalmic and Vision Research*,^[1]^ the name of the second author is written incorrectly as “Saeed Mohammadi" instead of “S. Saeed Mohammadi."
